# Therapeutic Potential of Experimental Stereotactic Hippocampal Cell Transplant in the Management of Alzheimer’s Disease

**DOI:** 10.3390/jcm14030891

**Published:** 2025-01-29

**Authors:** Loredana Mariana Agavriloaei, Bogdan Florin Iliescu, Robert Mihai Pintilie, Dana Mihaela Turliuc

**Affiliations:** 1 Department of Neurosurgery, “Grigore T. Popa” University of Medicine and Pharmacy, 700115 Iasi, Romania; mariana-loredana.curecheriu@umfiasi.ro (L.M.A.);; 2 Department of Neurosurgery, “Prof. Dr. N. Oblu” Emergency Clinical Hospital, 700309 Iasi, Romania

**Keywords:** Alzheimer’s disease, stereotaxic surgery, hippocampus

## Abstract

Due to a continuous increase in life expectancy and the progress made in specialized healthcare, the incidence of Alzheimer’s disease (AD) has dramatically increased to the point that it has become one of the main challenges of contemporary medicine. Despite a huge scientific and clinical effort, current treatments manage just a temporary alleviation of symptomatology but offer no cure. Modern trials involving cell transplantation in experimental animals require the involvement of neurosurgeons in the treatment protocol. CSF shunting, intraventricular infusions, or DBS for symptoms relief have been an integral part of the therapeutic arsenal from the very beginning. The development of stereotactic surgery has facilitated the experimental potential of cell transplantation in the hippocampus for Alzheimer’s disease. We conducted a narrative review of the literature in the top three medical databases (PubMed, Science Direct, and Google Scholar) using the keywords “Alzheimer’s disease”, “hippocampus”, and “transplant”. After eliminating duplicates, 241 papers were selected and screened by title and abstract. Two reviewers independently analyzed the 88 papers and chose 32 experiments that involved stereotactic hippocampal transplantation of cells in experimental animals with AD. The stereotactic transplantation of cells such as mesenchymal stem cells (MSCs), neuronal stem cells (NSCs), induced pluripotent cells (iPSCs), astrocytes, and derivates from stem cells was analyzed. The experiments used either a chemically induced or transgenic AD model and observed the impact of the stereotactic transplantation with behavioral testing, MRS spectroscopy, and biochemical analysis. The stereotaxic method delivers minimal invasive treatment option by cell transplantation at the hippocampus. The results showed that amyloid deposits were lower after transplantation, showing a positive impact. Other impactful results involve proliferation of neurogenesis, downregulation of anti-inflammatory response, and increased neuronal plasticity. The increased precision with which the stereotaxic method manages to target deep structures of the brain and the results of the reviewed papers could represent an argument for future human trials. More studies are needed to confirm the viability of the transplanted cells and the long-term effects.

## 1. Introduction

Alzheimer’s disease represents one of the main medical challenges of the modern world, with an increasing number of people being affected, due, in part, to a radical increase in life span as well as improved healthcare for the elderly. It is the fourth leading cause of disability-adjusted life-years in persons over 75 years [1]. Current treatments alleviate symptoms and are mostly pharmaceutical, but phase-1 and phase-2 studies conducted in animal research have been developed to understand the disease’s pathophysiology better and maybe find a cure in the process. The neuroscience research field has combined forces with neurology and neurosurgery to focus on curative treatment possibilities [2].

Alzheimer’s disease is characterized by a progressive decline of performance in everyday activities, with the development of apathy, depression, impaired communication, disorientation, poor judgment, and difficulty in swallowing and walking [3]. The cognitive decline is linked to the accumulation of amyloid-beta (Aβ) and tau proteins [4]. These pathophysiological changes, that start at the hippocampus, are the two main hallmarks of AD pathology [5]. Studies have shown that amyloid plaque deposits interfere with inter-neuronal communication, leading to impaired neuron function, neuronal death, memory loss, and abnormal behavior [6]. Neuronal communication deficit translates into a synaptic loss and defective neurogenesis, and its effects are devastating for the brain [7]. Neurogenesis is an important process that contributes to learning and memory, being a unique form of structural and functional plasticity located in the hippocampal dentate gyrus and subventricular zone of the lateral ventricles [7]. The synaptic loss seen in AD stops neurogenesis and promotes further neuronal loss.

Neurosurgical procedures offer the opportunity to direct treatment of pathophysiological features of a disease by minimizing adverse effects and bypassing the blood–brain barrier (BBB). Stereotaxis is a neurosurgical procedure first performed in the 1940s [8] that has significantly evolved and now has applications in both diagnostics, such as brain biopsies [9] and stereoelectroencephalography (SEEG) [10], and treatments like deep brain stimulation (DBS) and radiofrequency ablation [11]. In today’s technological era, stereotactic surgery presents an opportunity to transplant cells or deliver medication with minimal impact on the brain, offering possibilities for neuronal renewal or reducing the protein burden of β-amyloid and neurofibrillary tangles.

The present narrative review aims to synthesize our current understanding of the effects of hippocampal transplanted cells as a treatment for AD in experimental animals. We conducted an extensive search (PubMed, Science Direct, and Google Scholar) without publication date restriction until April 2024 for published articles containing the keywords “Alzheimer’s disease”, “hippocampus”, and “transplant”. After the first search, 241 papers were found, but only 88 studies remained after carefully analyzing the titles and abstracts. Two separate reviewers read the papers in full and found only 32 papers that were relevant to the present narrative review involving transplant methods for Alzheimer’s disease in experimental animals or had materials and methods explained to compare data. The summary of the reviewed articles can be seen in Table 1.

## 2. Results

### 2.1. Physiopathology of Alzheimer’s Disease

The pathological mechanism for AD is a complex multifaceted process, with several hypotheses that can be used to develop targeted treatment options. Two of the most studied hypotheses include the β-amyloid aggregation and the hyperphosphorylation of tau protein [42].

The β-amyloid implication is the most influential hypothesis [43]. The aggregation of β-amyloid peptides into insoluble fibrils and plaques initiates a cascade of pathological events. The insoluble aggregations are named amyloid plaques and accumulate in the gray matter of the brain, interfering with neuronal function and triggering a chronic neuroinflammatory process [44]. It is believed that the toxicity of β-amyloid leads to oxidative stress, mitochondrial dysfunction, and synaptic loss. This cascade ultimately contributes to widespread neuronal death and cognitive decline [42].

The tau hypothesis focuses on the role of tau protein in the formation of neurofibrillary tangles (NFTs), another hallmark of AD [45]. In AD brains, tau proteins undergo an abnormal hyperphosphorylation. This process causes it to dissociate from microtubules and aggregate into NFTs. These tangles are neurotoxic and impair intracellular transport, leading to neuronal dysfunction and death [46].

### 2.2. Animal Models Used in AD Research

**Figure 1:**
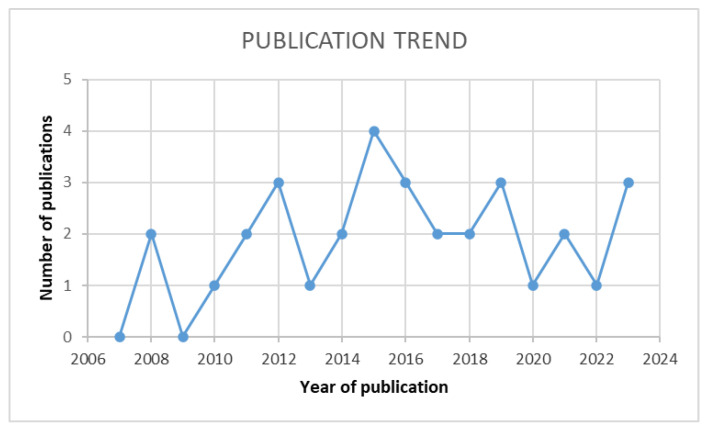
The trend of scientific publications relevant to the narrative review with a growth in 2010 and a spike in 2015.

Growing interest in developing new cell therapies with a direct impact on Alzheimer’s disease has been possible because of preclinical animal studies that take the ideas developed from in vitro effects seen on the pathological aspects of the disease to in vivo observation and research. The selected papers are ordered by year of publication in Figure 1 to emphasize the growing interest in this topic. The reviewed papers have used both mice and rats in their experiments, both lesioned [38] or induced models [6,17,31,32,33,34,35,36,37,39] or transgenic types of animals [6,7,12,13,14,16,18,19,21,22,23,24,25,26,27,28,29,30,38,40,41], showing that many AD animal models can be used to better define a new treatment. Comparing the type of experimental animals used in studies, the transgenic model was used by 21 papers in comparison with 11 research papers that used lesioned or pharmacological-induced AD models. In his review, Salari summarized the most known animal models for experimental studies, concluding that different animal models can be used to study different aspects of the disease and that depending on the researcher’s aim, they can select either a pharmacological-induced model or a transgenic one [47].

**Figure 2:**
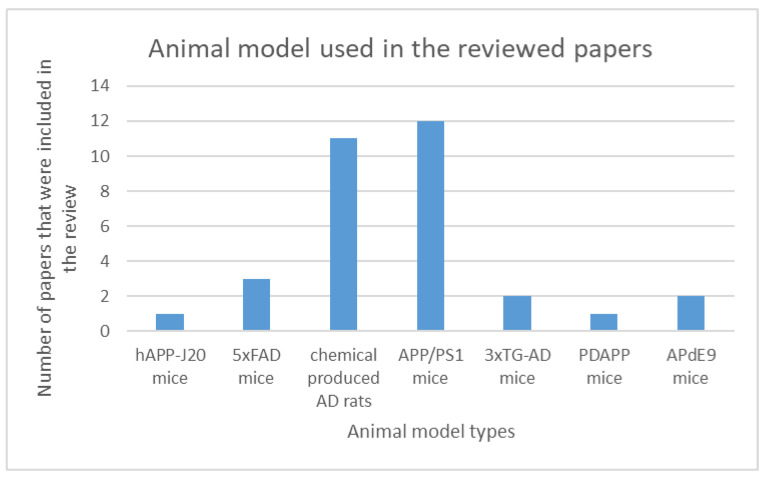
Animal models that were used by reviewed papers and the number of papers that included that type of AD model.

In Figure 2, we illustrate the number of papers that used either chemically induced [6,17,31,32,33,34,35,36,37,39] or transgenic models [6,7,12,13,14,16,18,19,21,22,23,24,25,26,27,28,29,30,38,40,41] in their experiments. The most common chemical used for inducing Alzheimer’s disease is amyloid-β. These experiments require a neurosurgical procedure to inject the chemical compound either intraventricularly or intrahippocampally. The administration routes have similar effects, and both are scientifically proven to induce Alzheimer’s disease with a single administration [48]. The main advantage of the β-amyloid model is that it manifests the pathological hallmarks of the disease, such as amyloid plaques, neurofibrillary tangles, and behavioral alterations [49].

The most used transgenic model is APP/PS1, used in 12 of the 32 reviewed papers. This animal model has the advantage that amyloid plaques have a similar morphology to humans, but the drawback is that the plaques develop over 3–4 months in the hippocampus and behavioral manifestations become evident at 7 months of age [48,49]. The 5xFAD transgenic model is the most advantageous because the amyloid plaques develop early, by 2 months of age, and the progression is the same regardless of the sex. The downside of this animal type is the reported hyperactivity that could potentially influence behavioral tests [50]. The LaFerla mouse (3xTg) is an animal model that develops both amyloid plaques and tau tangles, but these features become evident at 12 months of age, requiring long-term surveillance [50]. The PDAPP model is similar in terms of advantages to APP/PS1, developing similar pathological features with AD patients [49]. The hAPP-J20 mice model develops significant neuronal loss at 6 months of age, in addition to amyloid plaques that appear at 9 months [50].

Either transgenic or chemical-induced animal models have similarities with the human counterpart of Alzheimer’s disease. Therefore, the selection of the animal model used in the research was performed in line with the scope of the scientific aim. In our reviewed papers, models that exhibit both plaques and tangles were used to test a neurosurgical procedure and treat the burden of proteins that accumulate and determine the disease.

### 2.3. Neurosurgical Procedures for Alzheimer’s Disease

The field of neurosurgery has implications in finding a cure for neurodegenerative diseases; therefore, different techniques have been experimented with to help patients with their symptoms. In a review of neurosurgical methods for Alzheimer’s disease, the authors found human trials for CSF shunting, intraventricular infusion with cholinergic agents and neuroprotective factors, nerve growth factors, DBS, and even vague nerve stimulation, all with little or no effects [51]. All methods had little effect on the pathophysiological aspects of the disease, with impact only on symptoms. DBS is one of the methods used in both experimental studies on animal models and patients with AD. Studies that used DBS of the fornix have shown improvements in learning and memory performance [52,53,54]. Clinical trials investigated fornix/hypothalamic deep brain stimulation (DBS) for Alzheimer’s disease (AD) after results from experimental animals showed improvement. In A phase-1 clinical trial (NCT00658125), six patients with mild AD showed modulated hippocampal memory circuits, potential cognitive improvements, and restored glucose metabolism, with no serious adverse events [55,56]. A phase-2 clinical trial (NCT01608061) involved 42 patients, showing the safety of the procedure but no significant cognitive changes. Patients over 65 displayed some cognitive improvements [57,58,59].

The development of oral medication has seen an increasing focus on neurodegenerative diseases, but long-term use brings side effects and impacts on the quality of life of the patients. The need for minimally invasive surgery is clear to develop a high-impact procedure with low risks [60].

Recent developments in neurosurgical strategies and bioengineering of cells have fueled a new compound field of cell therapies for neurological disorders with specific cell targets representative of pathologies.

In the quest to find a cure for Alzheimer’s disease, researchers have developed an interest in experimenting with cell transplantation into the hippocampi of experimental animals. Beginning in 2006, the reviewed papers have grown in number, as depicted in Figure 1.

### 2.4. Stereotactic Surgery in Other Neurodegenerative Diseases

Stereotactic surgery has significantly advanced the precision and efficacy of neurosurgical procedures. In stereoelectroencephalography (SEEG), stereotactic techniques enable the accurate placement of electrodes deep within the brain to map epileptic foci in patients with drug-resistant epilepsy [61]. This precise targeting is crucial for identifying the seizure onset zone and guiding subsequent treatments, such as radiofrequency ablation or responsive neurostimulation. The major complications in SEEG are intracranial hemorrhage (pooled prevalence of 1.0%), infections (pooled prevalence of 0.8%), and mortality (pooled prevalence of 0.3%) [62].

For brain biopsies, stereotactic methods enable neurosurgeons to accurately target and obtain tissue samples from deep-seated lesions, aiding in the diagnosis of brain tumors, infections, and other abnormalities [63]. This minimally invasive approach reduces the risk of complications and improves patient outcomes [64]. The complications most commonly associated with this procedure are the onset of new deficits, seizures, infections, and hemorrhages, with mortality between 0% and 2.3% [9].

In deep brain stimulation (DBS), stereotactic techniques are used to implant electrodes in specific brain regions to treat movement disorders such as Parkinson’s disease, essential tremor, and dystonia [65]. DBS has been shown to alleviate motor symptoms and improve the quality of life for patients with these conditions [66]. The integration of advanced imaging techniques and robotic technologies has further enhanced the accuracy and safety of these procedures, making them valuable options for patients with complex intracranial conditions [66].

### 2.5. Different Cell Types Used for Transplant

Researchers have been testing on experimental animals and developing new treatment options for the hallmark pathological features of Alzheimer’s disease, thanks to the evolution of minimally invasive techniques of neurosurgery, such as stereotaxic surgery. The injection of different cells has been a particularly feasible option for the elimination of amyloid plaques or neurofibrillary tangles. Mesenchymal stem cells (MSCs) are an example, used for their proprieties of reducing neurological symptoms and promoting recovery of dementia disorders because of their immunomodulatory function and anti-inflammatory and anti-apoptotic response [27,34].

Bone marrow stem cells (BM-MSCs) are a self-renewing multipotent cell, derived from MSCs, with the propriety of differentiating into neural cells and secreting paracrine factors to enhance the regeneration of injured cells and decrease inflammatory response [17,35]. Another stem cell representative are neuronal stem cells (NSCs), which have the potential of self-renewal, and after transplantation, their advantage is increasing brain-derived neurotrophic factor (BDNF) [37]. Induced pluripotent stem cells (iPSCs) have been used as an alternative to embryonic stem cells, without ethical concerns and with advantages of bypassing donor cell rejection and without need for immunosuppressants [12].

Astrocytes are glial cells that have different normal functions, already located in the brain. One feature of the glia that can be stimulated by neurosurgical transplantation is amyloid-β clearance by degradation and phagocytosis [18,19].

#### 2.5.1. Mesenchymal Stem Cells

Mesenchymal stem cells (MSCs) are found in bone marrow and are known as pluripotent cells because of their ability to differentiate into specific cells such as blood cells, cartilage, bone, muscles, adipose tissue, and even neural cells (glia and neurons) [67,68]. Studies have shown that MSCs are hypoimmunogenic and they can help in the immune response by releasing cytokines and trophic factors [16]. Because of these factors, MSCs have been used in experiments for AD disease, with the primary endpoint of treating the neuroinflammation caused by amyloid plaques [39]. Cognitive testing was performed mostly with water mazes and with a T water maze conducted in week 9 post-surgery. The hippocampal transplant group significantly improved their time [16]. The same results were obtained in a study by Huang N et al. with tashinone-IIA-incubated MSCs that were transplanted and examined with the Morris water maze (MWM), showing better results on the fourth day of the trial [39]. Tashione IIA (TIIA) is a biologically active component [69] that was used to reduce neuroinflammation in transplanted cells [39]. In a study that compared transplanted MSCs with NSCs for AD treatment, cognitive testing was performed by the open field test (OF), which showed a superiority for neural stem cells in restoring behavior to control levels, and the novel object recognition (NOR) test, which showed improvement in cognitive behavior but no significant difference between transplanted groups [27].

The question of whether the cells remain in the transplanted area or migrate towards other parts of the brain was raised and answered by experiments that showed that MSCs remain clustered near the injection site [16]. As predicted by earlier studies, MSCs decrease the number of amyloid-β plaques compared to the transgenic model without transplant (APP/PS1), showing the benefits of MSCs in clearing plaques [27]. The immunomodulatory capacity of the MSCs was observed and compared with TIIA-MSCs by measuring the levels of IL-1, IL-4, IL-10, and TNF-α. The results show that both groups with transplanted cells (TIIA-MSCs and MSCs) displayed attenuated interleukin levels, demonstrating their ability to regulate neuroinflammation, with a biphasic regulatory capacity to suppress the immune response when it is too strong or promote an immune response in case of weak inflammation [39].

Based on the positive results of injecting MSCs in animal hippocampi, an experimental study was conducted to evaluate if different doses could have distinct protective effects [34]. The study compared a low dose of 25 × 10^4^ MSCs to a high dose of 50 × 10^4^ and concluded that the lower dose has a protective effect on the brain in terms of oxidative stress injuries and causes a reduction in TNF-α with an increase in IL-10, therefore having a beneficial effect. The high-dose group showed a reduction in the therapeutic inflammatory effect and insignificant variation in the spatial learning function when compared to the AD control group, leading to the conclusion that a higher dose of MSCs does not increase their protective effects and may even reduce them [34]. The same group aimed to test preconditioned MSCs with dimethyl fumarate (DMF) after transplantation in AD animals, trying to enhance the cell’s therapeutic efficacy [36]. MSCs were incubated with DMF (20 µM) for 24 h, and afterwards, 25 × 10^4^ cells were transplanted into the hippocampi of rats with chemically induced AD.

In comparison with transplanted MSCs, the DMF preconditioned ones showed a substantial effect on cognitive performance. The experiment followed the preconditioned MSCs in vitro and demonstrated that they enhance the survivability, proliferation, and antioxidant capability of the cells [36]. Another key feature of MSCs is neurogenesis, demonstrated for adipose-derived mesenchymal stem cells (ADSCs) in vitro by Kang et al. [70] and in vivo by Yan et al. [21]. The idea for the animal experiment was to investigate if intrahippocampal transplant of ADSCs could promote neurogenesis for transgenic AD mice (APP/PS1 model). Immunofluorescent staining and co-labeling with bromodeoxyuridine (BrdU) and doublecortin (DCX) neuron markers resulted in ADSC transplantation-enhanced neurogenesis in the subgranular zone [21].

Microglia represent an important immune effector cell in the immune surveillance and tissue maintenance of the CNS, having implications in AD by surrounding amyloid plaques and infiltrating the deposits with their processes [71]. Knowing this, Ma et al. posed the question of if ADSC transplant could impact and enhance microglial activation and promote their ability in neuroinflammation in AD [20]. After hippocampal transplantation of ADSC and antibody Iba-1 labeling for activated microglia, they concluded that the transplant activated microglia in the region. Confocal microscopy demonstrated that Iba-1-positive microglia were near the amyloid plaques and had neuroprotective effects [20].

Another derived mesenchymal stem cell used for hippocampal transplantation is dental pulp stem cells (DPSCs) because of their availability and neurogenic differentiation potential. Researchers observed DPSCs differentiated into neuron-like cells that expressed neuron-associated proteins. The experiment showed a cognitive improvement after DPSC transplantation [6].

Due to the risks of rejection after transplantation, the possibility of using stem cells from fetal origin was tested with human amniotic mesenchymal stem cells (hAM-MSCs). hAM-MSCs have lower immunogenicity and immunomodulatory effects [72,73,74] but at the same time have the same anti-inflammatory effects that are thought to manipulate the neuroinflammation in AD pathology [36]. Cognition was tested using the NOR test and showed that hAM-MSCs improve learning and memory impairments. The pathophysiological effect was noted with a significant decrease in amyloid-β deposits and an inhibitory effect of plaque formation [24].

#### 2.5.2. Bone Marrow Stem Cells

Studies using lesions in animals with ibotenic acid bilaterally into the nucleus basilis magnocellularis (NBM) tested the cognitive impact of bone marrow stem cell transplantation [35]. Ibotenic acid is an amino acid found in certain mushrooms that researchers use to create lesions and induce neural damage in experimental animals [75]. The injected animals exhibit deficits in learning and spatial memory; therefore, it can be used as a model for studying AD [76]. Bone marrow stem cells were used for their property to differentiate into multiple cell types, for example, neural cells [77,78,79]. Bilateral hippocampal transplantation of cells was performed, and animals were observed in the Morris water maze test to evaluate their behavior after the surgery and two months afterward. The experiment indicated that transplantation attenuated Ibo-induced learning and memory impairment. The latency was improved by approximately 22.7%, having a mean latency to the platform before the experiment of 37 ± 1.5 s and an improved time of 28.6 ± 2.4 s after the experiment [35].

The cognitive aspect of AD is believed to be correlated with the decline of ChAT (choline acetyltransferase) activity [80], and theory was tested by injecting BMSCs (bone marrow stem cells) and NGF-BMSCs (gene-modified bone marrow stem cells) into the hippocampus of a rat model with Alzheimer’s disease [80]. NGF is an important trophic factor for neurons in the CNS that rescues injured cholinergic neurons. The authors compared not only control AD groups but also BMSC transplants and BMSC-NGF transplants. Cognitive improvement was seen in both transplanted groups, with significantly more effects in BMSC-NGF transplantation. NGF-positive immunostainings were observed in the BMSC-NGF group, indicating that the transplanted cells could differentiate into ChAT-like neurons and improve the decline produced by AD [80].

As stem cell therapy evolves, bone marrow stromal cells (GFP-BMSCs) are highlighted because the cell can serve as a stem cell reservoir for mesenchymal cells. GFP-BMSCs were transplanted into rat hippocampi and evaluated cognitively and histologically after 3 weeks. Cognitive improvement was shown with the Morris water maze test. Histology revealed a significant reduction in amyloid-β deposits in the treated group compared to the control, proving their potential for AD treatment [23].

#### 2.5.3. Neural Stem Cells

Neural stem cells (NSCs) are CNS progenitor cells with the capacity to self-renew and differentiate into neuronal or glial phenotypes [81], therefore with a utility in CNS injuries, such as neurodegenerative diseases. The cells can be transplanted after being isolated from different parenchymal structures (subventricular zone, hippocampus, olfactory bulb), or they can be genetically manipulated to express trophic factors [38].

EPI-NCSCs (epidermal neural crest stem cells) are multipotent NSCs (neural stem cells) that were differentiated into generating cells that expressed markers for neurons, glial cells, and others [33]. The idea was to transplant EPI-NSCs in the hippocampi of animals with AD in order to generate neurons or cholinergic neurons [33], as studies with bone marrow stem cells transplant proved [80]. After analysis in vitro of EPI-NSCs, it was proved that the multipotent cells can generate all neural lineages, and transplantation was performed. The transplantation group showed an improvement in behavioral alteration and performed better in the Y maze test. The histological procedures evoked an increase in cell numbers at the transplantation location, but by using the double-staining procedure, they proved that cells presented BrdU-GFAP and BrdU-ChAT, therefore differentiating cells into cholinergic neurons [33]. Another derived neural stem cell used in experiments was HuCNS-SCs (human neural stem cells, derived from donated fetal brain tissue), used in the context of neuronal restoration. Even though transplanted cells helped improve the cognitive function of mice, no impact was found among amyloid plaques or tau proteins after histological analysis, suggesting that the effect is only at the synaptic level [13].

OBNSCs-hNGF (adult human olfactory bulb neural stem cells over-expressing human nerve growth factor) are genetically modified NSCs that can lead to increased neuronal survival; this theory was proven by a team that transplanted the cells and found that after 8 weeks the cells not only survived but increased by 1.89-fold and resulted in cognitive improvement. The study concluded that the transplanted cells rescued damaged cholinergic neurons, prevented the progressive loss of neurons, and induced a regenerative response in hippocampal neurons [38]. Similar effects were found after NSC transplantation in triple transgenic mice, with an increase in neuron numbers [28]. Another similar study with NSC transplanted hippocampi cells proved that after transplantation, cells differentiated into astrocytes and neurons, and in addition their impact on cognitive performance, there was an increased level of cholinergic proteins and cholinergic neurons in the basal forebrain [26].

NSCs were also transplanted with a designer self-assemble peptide (DSP) which contained one functional domain Tyr-Ile-Gly-Ser-Arg (YIGSR) derived from laminin with the purpose of promoting survival and neuronal differentiation. The study results showed a decrease in apoptotic cells after transplantation with NSC-DSP in comparison with a simple peptide (SP), indicating a new approach to hippocampal transplantation [37].

The only in vivo exploration of metabolic changes is magnetic resonance spectroscopy (MRS), and NAA (N-acetyl aspartate) was linked to having a decreased peak in AD patients [82]. An experiment using hippocampi transplantation of NSCs (neural stem cells) for AD treatment was performed before and 6 weeks after the intervention with MRS of the subjects. The study showed an increased level of both NAA and Glu (glutamate) levels in the transplanted group, compared with controls, showing a benefit of the treatment [29], proving that MRS is a useful tool in this research area. Similar results were seen in two more studies, where NAA and Glu levels increased after NSC transplantation, showing an improvement in neuronal metabolic activity [7,25].

Another study that transplanted a modified line of human cortex-derived neural stem cells (NSI-HK532-IGF-1) detected a decrease in plaque load in both hippocampal and cortical brain regions. The effects were linked to an increase in microglia numbers with a neuroprotective phenotype that can assist in clearing toxic amyloid aggregates [30]. Studies on human neural stem cells transplanted into animal hippocampi showed similar results. A study that used iNPCs (human-induced neural progenitor/stem cells) from peripheral blood cells demonstrated differentiation into astrocytes and survived 12 months after transplantation. Chen et al. transplanted also hNSCs with similar results regarding microglial differentiation and synaptic improvement, but with no effects on amyloid deposits [15].

#### 2.5.4. Induced Pluripotent Stem Cells

IPCs (induced pluripotent stem cells) represent a step forward in research on degenerative CNS diseases due to their regenerative potential, with several studies researching Parkinson’s disease [83,84,85,86], Huntington’s disease [87,88], Multiple Sclerosis [89], amyotrophic lateral sclerosis [90,91], and AD [12,41]. The AD model used fibroblasts to generate iPSCs, as an autologous source that bypasses complications due to immune rejection and avoids immunosuppressant use. IPSCs were processed into a monolayer of self-renewing NPCs, called iPSC-NPCs [12]. ELISA quantification measured soluble and insoluble amyloid-β in both transplanted and control groups and revealed that iPSC-NPC hippocampal transplantation prevents amyloid-β deposits and/or favors the clearance of amyloid-β [12] in AD transgenic mice.

Another experiment performed on transgenic mice was that of Fujiwara et al., which used hiPSCs (human induced pluripotent stem cells) and therefore used immunosuppression (dexamethasone before transplantation and cyclosporine after transplantation daily). hiPSCs were harvested, and by the 8th day, the cells demonstrated nestin expression with a similar phenotype, with the possibility of differentiating into both cholinergic and GABAergic neurons. These more mature neuronal phenotype cells were transplanted into the hippocampi of transgenic mice and examined. Cognitive tests were performed and showed restoration of spatial memory function. Approximately 1 month after, immunohistochemical staining showed that hiPSC-derived neurons altered the differentiation of mouse neural stem/progenitor cells, increasing ChAT-positive neurons [41].

#### 2.5.5. Neuron and Neuron-like Cells

NSCs could differentiate into cholinergic neuron-like cells (CNLs) with the help of retinoic acid (RA) and nerve growth factor (NGF) to restore cholinergic function. RA is a naturally potent form of vitamin A that can enhance the cholinergic phenotype in neurons and other cells, while NGF is a trophic factor for cholinergic neurons [92]. Hippocampal transplantation of NSCs was performed after in vitro NSC culture with RA and NGF. Fluorescent staining with green fluorescent protein (GFP, protein marker) revealed that implanted NSCs were able to differentiate into neurons. The animals were analyzed in vivo for three months, and afterwards, they were sacrificed to see the survival rate and migration pattern of the transplanted cells. GFP-NSCs differentiated into CNLs and were mainly found in the hippocampus, with confocal microscopy revealing that the cells exhibited cholinergic neuron function. As in other studies, cognitive function was ameliorated with no significant difference in the total number of amyloid-β plaques [22].

Human umbilical cord mesenchymal stem cells (hUC-MSCs) are different from MSCs because of their feature of abundant tissue sources, and they are an easy method for separation and purification in experimental studies. Because of their fetal origin, the cells can secrete various neurotrophic factors such as brain-derived neurotrophic factor (BDNF), nerve growth factor (NGF), glial cell-derived neurotrophic factor (GDNF), and fibroblast growth factor-2 (GFG-2) [93,94,95]. BDNF is implicated in neurogenesis and synapse formation, inducing differentiation of neural stem cells into neurons and promoting their maturation [96]. To study the implication of hUC-MSCs with the amplification of BDNF, Hu et al. modified and evaluated BDNF-modified hUC-MSC-derived cholinergic-like neurons. Hippocampal transplantation of the cells was performed, and analysis showed that the BDNF-modified hUC-MSC-derived cholinergic-like neurons improved cognitive function (with spatial learning and memory ability scores higher than in the control group) [31].

AD pathology revealed that the hypofunction of Nav 1.1, a voltage-gated sodium channel subunit predominantly expressed in interneurons, was correlated with a dysrhythmic network that led to cognitive decline [97]. In their original study with transplanted interneurons derived from the embryonic medial ganglionic eminence (MGE) overexpressing Nav 1.1, Martinez-Losa et al. demonstrated that the new interneurons integrate into neuronal circuits and mature into functional inhibitory and regulatory interneurons [40].

#### 2.5.6. Astrocytes

In two serial studies published by Rea Pihlaja et al., the researchers investigated the potential of cultured mouse astrocytes to degrade amyloid-β deposits in the hippocampi of old transgenic mice [18,19]. In the first experiment, the research group prepared adult and neonatal astrocyte cultures for comparison purposes that were transplanted into the hippocampi of APdE9 and wild-type mice. The astrocytes were prepared and injected bilaterally in 2 μL at the speed of 0.25 μL/min in the CA3 area of the hippocampal formation following stereotaxic coordinates. In total, the experiment followed 14 transgenic APdE9 mice and 7 wild-type mice as a control group that were sacrificed at 1, 3, and 7 days post-transplant for further examination. As a result, no difference was found between adult and neonatal transplanted astrocytes in migration pattern or the ability to localize near amyloid-β deposits at 1, 3, or 7 days after transplantation, suggesting that the glial cells that were used rapidly recognize and associate with the amyloid deposits. The second endpoint of the study was to analyze and calculate the rate of internalization of amyloid-β in vivo, and this was found to be, on average, 44% of the adult astrocytes and 19% of the neonatal astrocytes [19]. Based on their findings, another study was conducted to determine whether transplanted adult astrocytes are also able to degrade and internalize amyloid-β deposits during a 2-month period.

In the first study, the authors reported that a downside of using a small number of animals was the fact that the results were not statistically relevant; therefore, in the next experiment, they conducted the transplant in 47 APdE9 transgenic mice and 45 sham operations in wild-type age-matched mice. They transplanted only adult astrocytes because of their better internalization into CA3 hippocampi of mice at two different ages, 12 months when the amyloid-β load is progressing fast and 23–24 months when the amyloid-β burden has already been saturated. The sacrificed time points were at 2 weeks, 1 month, and 2 months. The results suggested that at the 2-week follow-up, the astrocytes were found in large clusters, but as time passed, they began to dissociate from each other and started to be attracted by amyloid-β deposits. Regarding amyloid-β reduction by glial cell transplant, the study observed a 76% reduction in amyloid-β burden in 12-month-old transplanted mice and a 41% reduction in 23–24 month-old mice, concluding that astrocytes managed to produce a greater reduction in amyloid-β burden when the pathology was still in development [18]. These two studies concluded the beneficial impact of astrocyte transplant, in regard only to the pathophysiology of the disease and amyloid-β clearance, without the clinical aspects of the disease being examined.

## 3. Discussion

Stereotaxic neurosurgery represents a minimally invasive option for specific targeting of the affected areas of Alzheimer’s disease. This direct treatment option can help develop new techniques that include the neurosurgical field. The stereotactic identification and transplantation of cells minimizes adverse effects, in comparison with more invasive neurosurgical techniques used in the past (for example, shunt procedures), and bypasses the blood–brain barrier.

Almost all experimental designs reviewed performed behavioral tests pre- and post-transplantation to quantify the real-life impact. The most used test was the Morris water maze (MWM), a complex test with a spatial learning part for rodents that is assessed across repeated trials and a reference memory part determined by preference for the platform area when the platform is absent [98]. Other behavioral tests used were novel object recognition (NOR), the open field test (OFT) and Y-/T-mazes. All experiments concluded that cell transplantation had a significant impact on cognitive function and improved scores in all conducted tests. The studies showed that cell transplantation, regardless of the nature of the cell type, will result in restoration or at least improvement of brain function.

MRS (MRI spectroscopy) can be used to determine improvement in brain metabolism after transplantation, without harming the animals; therefore, it can be a method for long-term follow-up and can help reduce the quantity of sacrificed mice. The imaging technique offers a sensitive and non-invasive method of quantitative measurements regarding treatments. Previous studies have established that NAA (N-acetyl aspartate) and Glu (glutamate) were found to decrease in AD, while mI (myo-inositol) was found at a higher level in AD animals [99]. NAA and Glu peaks were elevated, while mI decreased in the brains of AD animals treated with hNSC transplantation, showing a benefit for this type of cell [25]. Similar results were found after 6 and 10 weeks with NSC transplantation, showing a statistical difference when compared to the sham transplantation group [7,29]. Zhang W et al. quantified the increase in NAA and Glu by 1.43- and 1.27-fold in comparison with sham operation and control groups.

Paraclinical analysis offers an alternative investigation to invasive techniques for monitoring AD. ADSC, hAM-MSC, and MSC transplantation were monitored by expression of pro-inflammatory cytokines (TNF-α and IL-1β) and anti-inflammatory cytokines (IL-4 and ARG1), with a resulting process of reduced pro-inflammatory response and elevated levels of anti-inflammatory markers [20,24,34].

The most impactful result followed was amyloid-β deposits because of their pathophysiological aspects regarding AD evolution and their impact on cognitive impairment. After ADSC transplantation, the researchers observed that microglial activation resulted in a decreased load of amyloid plaques, indicating its connection [20]. Astrocytes show a similar result on amyloid internalization and reduce the plaque load regardless of being adult or neonatal astrocytes that have been transplanted [19]. Pihlaja et al. demonstrated hippocampal astrocyte transplantation in mice of different ages in another study and showed that amyloid burden decreased by 70% in 12-month-old mice and 40% in 23–24-month-old mice [18].

BMSC transplantation also obtained a reduction in amyloid plaque deposition of treated mice [23]. Amyloid plaque showed a reduction in AD mice brains after IPSC-NPC and hNSC transplantation by biochemical measurements of soluble and insoluble amyloid-β [12,25]. For the hNSCs group, the decreased level of amyloid did not reach significance, showing that the effect only alleviated the burden [25]. Quantitative image analysis showed that hAM-MSC transplantation decreased amyloid deposits by attenuating amyloid levels in both the frontal cortex and hippocampus [24]. ELISA analysis of the whole brain demonstrated a 50% reduction in amyloid load after human NSC transplantation [30].

Amyloid plaques located in the fornix and subiculum were impacted by MSC transplantation, and plaque load did not differ significantly from the control group without AD [16], proving a restoration of brain architecture before AD impact. Both MSC and NSC transplantation impact the deposits of amyloid in the hippocampus [27].

Activated microglia is linked to neuroinflammation in some instances, but recent experiments have demonstrated its capacity to mediate the clearance of amyloid-β. A high level of activated microglia has been observed in experimental designs after ADSC transplantation, with a neuroprotective effect and impact on amyloid-β clearance [20]. Microglia differentiation was shown by the presence of marker Iba-1 after transplantation with BDNF-modified hUC-MSC-derived cholinergic-like neurons [31], hAM-MSCs [24], NSCs [27], and human NSCs [30]. These data suggest that NSCs promote a different phenotype of microglia after direct correlation with amyloid plaque numbers after transplantation.

ADSC, hAM-MSC, EPI-NCSC, hNSC, and NSC transplantation resulted in increased neurogenesis that differentiated into neurons in the subgranular zone of the dentate gyrus, proven by BrdU, DCX, and Nestin expression [7,15,21,24,25,26,29]. Hippocampal transplantation with NSCs increased the number of neurons to a level similar to the control group, indicating that the impaired neurons were repaired or that a high population of new neurons was generated [28,29]. Increased neurogenesis and differentiation into cholinergic-like neurons was also seen in BMSC-NGF transplantation, indicating that NGF might play an important role in restoring learning and memory function [25,33]. Neuronal differentiation of transplanted NSCs can be facilitated and protected by administration of cells in a DSP (designer self-assemble peptide) by preventing transplanted cell apoptosis when compared to a SP (self-assemble peptide). The mechanism of action is believed to be through upregulation of p-Akt and downregulation of cleaved caspase and by balancing the ratio of Bcl-2 to Bax proteins involved in neuronal apoptosis [37]. Stereological studies after OBNSC transplantation demonstrated a significant increase in neurons at the injection site, but after quantification, the researchers proved that after 8 weeks, there was a 1.89-fold increase in cells compared to the initial cell populations transplanted [38]. Another group of researchers proved that BM-MSC transplantation induced neuronal plasticity and increased neuronal survival by demonstrating an increase in ERK phosphorylation and CREB, two factors of the MAPK-ERK pathway critical in synaptic plasticity signaling [17]. Neuron-associated proteins such as DCX, NeuN, and NF200 were tested after DPSC transplantation and revealed a higher number compared to the sham operation or control group, indicating an improvement in cell function [6].

The question of impact has been proved by the reviewed studies, with different effects on cognition or pathological aspects of the disease. The remaining aspect of these studies is the viability of the cells and if the burden of amyloid plaques will remain low or increase after the transplanted cells disappear. Table 1 summarizes the impact of transplanted cells and the viability followed by the research, not only showing that at 12 months the cell remains viable but that the cells integrate though neurogenesis and have an impact on the evolution of the disease. The earliest documentation of cell viability after transplantation is at 5 days [20], validating the experimental design and location of stereotaxic trajectory followed by Paxton’s atlases. The longest experiment was conducted over a 12-month period and concluded that not only do cells remain viable but they differentiate into neural cell lines (astrocytes, microglia, and neurons), having an impact on the cytoarchitecture of the affected brain [15].

An indirect result of growing cholinergic levels has been shown by transplantation of BMSCs into lesion-induced AD mice, with results seen in learning and memory testing [35]. Cholinergic increased significantly three months after NSC transplantation, which was previously performed by a preinduction stage using RA and NGF. The process of differentiation and induction resulted in CNL (cholinergic neuron-like cells) [22]. Another group of cells that have been differentiated in vitro and then transplanted is BDNF-modified hUC-MSC-derived cholinergic-like neurons that resulted in high cholinergic levels. hIPSCs are another example of transplanted cells that expressed ChAT and induced ChAT-positive neurons that were distributed through the overlying cerebral cortex around the injection site [41]. Hippocampal transplantation of IPSC-NPCs improved synaptic activity and function; an effect revealed by electrophysiological experiments that show a lower I-O curve in fEPSP-evoked potentials [12]. Synaptic connectivity and density can be measured by proteins like synaptophysin and synapsin, which were elevated after HuCNS-SC and NSC transplantation [7,13]. Significantly increased levels of ChAT proteins and ChAT-positive neurons were found in the basal forebrain of mice transplanted with NSCs [26].

Oxidative stress has been a hallmark in the pathology AD; therefore, possible treatment of the disease by reducing oxidative stress has been studied. ADSC transplantation reduced the level of oxidative stress compared to control animals injected with HBSS [21]. Similar results were seen in a study that compared lower vs. higher doses of MSC transplantation, and it was concluded that the therapeutic response of reducing oxidative stress was only seen in the lower dose group [35]. Preconditioning MSCs before transplantation with DMF (dimethyl fumarate), which has an anti-oxidative stress effect, decreases its levels at the injection site [36]. BM-MSCs exhibit a similar response of lowering oxidative stress levels only 11 days after transplantation [17].

Stem cell transplantation can result in complications for the host such as malignancy development, biocompatibility issues, or unwanted immune response [100]. Stem cells share characteristics with cancer cells, such as resistance to apoptosis and the capacity for reproduction; therefore, implantation with stem cells could increase the risk of developing cancer [100,101].

Using human-derived cell lines in animal experiments brings into question the rejection of the transplanted cells. To prevent rejection by the host, researchers have used immunosuppression before and after transplantation. In preparation for transplantations, dexamethasone [41] and cyclosporine [38] have been administered. Transplantation regimes differ from research to research, with some studies using daily administration of cyclosporine [38,41] and doxycycline [12]. Another regime for immunosuppression was intraperitoneal injection of anti-LFA-1, anti-CD40L, and h-CTLA-4-Ig fusion protein on the day of the experiment and on the 2nd, 4th, and 6th day after transplantation [13,14]. Researchers who transplanted human NSCs administered mycophenolate for 7 days post-transplantation and tacrolimus for another 10 weeks before euthanasia [30]. In one study, the experiment was conducted on immunodeficient Foxn1 mice, therefore not necessitating additional immunosuppression [15]. No tumors or diseases were reported in the experiments that used immunosuppression, but it is a problem to consider before planning such a trial.

## 4. Limitations

The present study has the limit of being a narrative review due to the high variability in the transplanted cells and the experimental designs in the reviewed papers. However, a review is needed to summarize the actual knowledge of the implication of stereotaxic treatment for Alzheimer’s disease.

This narrative review analyses the cognitive and pathological implications of stem cell transplantation into the hippocampus by stereotactic implant. The studies were conducted on rats and mice, and while both are rodents that are used in experimental research, the comparisons need to be assessed bearing in mind that there are differences between these two species in terms of how they evolve in the AD setting and how they respond to treatment or surgery itself.

Another pitfall would be the fact that the experiments used chemically induced (one study even used a lesioned model for AD) and transgenic models. While the trend is using transgenic animals that can behave more like a human counterpart with AD, we believe that the chemical-induced model is a strong experimental base that requires a lower budget and represents the foundation for what is to come in neuroscience.

The cell quantity injected differed throughout the studies, showing that even smaller quantities of transplantation will influence the AD model, as mentioned above. The take-home message is that too many transplanted cells can have no impact or even maybe a negative impact [35]. The difference between injected cells demonstrates the idea that a minimum of 1 × 10^5^ cells need to be injected in order to have an impact.

## 5. Conclusions

Even though neurosurgery does not represent a first option for Alzheimer’s disease, the reviewed papers demonstrate a positive impact and a viable option with few adverse effects. The minimally invasive technique is currently used in neurosurgical practice and can be implemented as a mainstream treatment for cell transplant.

Going further into developing a human clinical trial with either cell line for hippocampal transplantation requires the assurance that cells will survive beyond a period and the benefits will outweigh the risks and morbidity of surgery. As mentioned before, the hallmark for now is a 12-month survival rate after observation. Some suggest that this is not important because transplanted cells differentiate into cells of neural lineages; therefore, the survival rate of the transplanted cells is not an important factor [102].

All reviewed papers determined an improvement in AD treatment by stereotactic hippocampal transplantation of either experimental cell. Clear patterns of effects can be seen by transplantation of different cell lineages, concluding that further studies are needed to see which cell would have the most impact in a human trial. By testing cross-racial cells, the authors not only proved a decrease in amyloid deposits but also an increase in synaptic activity, proving that even with the need for immunosuppression and its side effects, the benefits of transplantation are real.

The methodology of the experiments seems to have reached its peak, with papers that followed up after 12 months, and can be of help in conducting such studies. Further studies need to consider the discrepancies between transplanted cell quantity and the period of examination after the procedure. These are important steps in harmonizing scientific information regarding a new minimally invasive method for the treatment of such a vulnerable population, who are given a sentence of memory death at diagnosis.

## Figures and Tables

**Table 1:** Reviewed articles sorted by type of animal used, documenting the transplanted cell type into the hippocampus, cell count, histological results, and maximum documented survival of transplanted cells.

First Author (Year) [Ref]	Animal Model	Experimental Groups	Type of Transplanted Cells	Cell Count	Outcomes	Duration of Cell Survival	Test/Duration	Test Results
Armijo E et al. (2021) [12]	3xTG-AD transgenic mice	4 groups: 7 × WT + PBS; 7 × WT + iPSC-NPCs; 7 × 3xTg; 7 × 3xTg + iPSC-NPCs	iPSC-NPCs	5 × 10^5^ cells/mL	decreased amyloid plaque deposits, improved synaptic activity	2 months	(1) OLT; (2) Barnes maze	(1) significant improvement in performance, (2) ability to learn the task quicker
Ager RR et al. (2015) [13]	3xTG-AD; CaM/Tet-Dta mice	2 groups: 6 × HuCNS-SC; 6 × WT	HuCNS-SC	1 × 10^5^ cells/mL	increased the levels of synaptic and growth-associated proteins	6 weeks	(1) MWM (2) NOR/10 min	(1) stronger memory formed, (2) significantly more time with the object placed into a new context
Chen KS et al. (2023) [14]	5xFAD transgenic mice	4 groups: 10 × WT; 10 × 5xFAD; 9 × 5xFAD − vehicle-injected; 10 × hNSC	hNSC	3 × 10^5^ cells/mL	improved synaptic activity	35 weeks	MWM/9 days	restored learning and memory and recapitulated the learning curve of WT animals, which did not differ significantly in the latency to the platform
Zhang T et al. (2019) [15]	5xFAD transgenic mice	3 groups: 6 × hiNPCs; 6 × 5xFAD; WT	hiNPCs	2 × 10^5^ cells/mL	increased synaptic transmissions	12 months	(1) Y-maze task; (2) Barnes maze.	(1) significantly decreased alternation frequency and exhibited a markedly improved performance, reaching the level of WT mice, (2) exhibited gradually and significantly shorter latencies
Matchynski-Franks JJ et al. (2016) [16]	5xFAD transgenic mice	7 groups: 8 × lateral ventricle, 8 × hippocampus, 8 × hippocampus + lateral ventricle, 8 × sham operation control, 8 × WT, 8 × AD sham operation, 8 × 5xFAD	MSC	2 × 10^5^ cells/mL	decreased amyloid plaque deposits	10 weeks	T-radial-maze	lateral ventricle and hippocampal groups significantly improved on trial block
Lee JK et al. (2010) [17]	AD mice with amyloid injection	2 groups: BM-MSC; control group	BM-MSC	1 × 10^5^ cells/mL	increased neuron numbers, reduced oxidative stress	11 days	MWM/3 days	capable of improving memory impairment
Pihlaja R et al. (2011) [18]	APdE9 transgenic mice	2 groups: 47 × astrocytes, 25 × WT with sham operation	astrocytes	4 × 10^4^ cells/mL	adult and neonatal astrocytes decreased amyloid plaque load and did not migrate from the injection site	7 days	-	-
Pihlaja R et al. (2008) [19]	APdE9 transgenic mice	2 groups: 14 × astrocytes, 7 × WT	astrocytes	1.25 × 10^5^ cells/mL	decreased the amyloid burden more in younger transplanted mice, compared to older animals	2 months	-	-
Ma T et al. (2013) [20]	APP/PS1 transgenic mice	3 groups: 10 × HBBS; 10 × ADSC + HBBS; 5 × WT	ADSC	1 × 10^5^ cells/mL	decreased amyloid plaque load by activating microglia	5 days	(1) MWM/5 days; (2) NOR/10 min	(1) similar results to the WT group, (2) showed more curiosity about the novel object
Yan Y et al. (2014) [21]	APP/PS1 transgenic mice	2 groups: 5 × HBBS; 5 × ADSC	ADSC	1 × 10^5^ cells/mL	improved neurogenesis and neuronal differentiation and reduced oxidative stress	not mentioned	NOR/10 min	interacted more with the novel object than with the familiar one
Gu G et al. (2015) [22]	APP/PS1 transgenic mice	3 groups: 25 × CNL; 25 × PBS; 50 × WT	CNL—cholinergic neuron-like cells	1 × 10^5^ cells/mL	improved synaptic activity	3 months	MWM/4 days	decreased latency in identifying the hidden platform compared with the control group
Wen SR et al. (2013) [23]	APP/PS1 transgenic mice	2 groups: 6 × GFP-BMSC; 6 × sham operation	GFP-BMSC	2 × 10⁷ cells/mL	decreased amyloid plaque load	3 weeks	MWM/5 days	significantly better on the water maze than the sham operation group
Zheng XY et al. (2017) [24]	APP/PS1 transgenic mice	3 groups: 10 × APP/PS1, 10 × hAM-MSC, 10 × WT	hAM-MSC	1 × 10^6^ cells/mL	decreased amyloid plaque load, microglia activation, reduced pro-inflammatory markers, increased neurogenesis	2 months	(1) MWM/5 days (2) NOR/10 min	(1) significantly shorter latencies, (2) transplanted mice significantly improved the behavioral deficits
Li X et al. (2016) [25]	APP/PS1 transgenic mice	3 groups: 12 × hNSC, 12 × PBS, 12 × WT	hNSC	1 × 10^5^ cells/mL	increased synaptic activity, decreased amyloid deposits	6 weeks	-	-
Zhu Q et al. (2020) [26]	APP/PS1 transgenic mice	3 groups: 12 × PBS, 12 × NSC, 12 × WT	NSC	1 × 10^5^ cells/mL	cell differentiation into astrocytes, neurons	2 weeks	MWM/4 days	shorter latencies
Campos HC et al. (2022) [27]	APP/PS1 transgenic mice	4 groups: 15 × WT, 15 × APP/PS1, 15 × MSC, 15 × NSC	NSC—neural stem cell; MSC—mesenchymal stem cell	4 × 10^5^ cells/mL	decreased amyloid plaque deposits, only NSC increased microglia activity	not mentioned	OF test	NSC grafting was able to restore locomotion to control levels
Chen SQ et al. (2014) [28]	APP/PS1 transgenic mice	3 groups: 10 × NSC; 10 × PBS; 10 × WT	NSC	1 × 10^6^ cells/mL	increased neuron numbers	8 weeks	MWM/6 days	significant overall effect on escape latency
Chen SQ et al. (2012) [29]	APP/PS1 transgenic mice	3 groups: 15 × NSC, 15 × PBS, 15 × WT	NSC	1 × 10^6^ cells/mL	increased neuron numbers	8 weeks	-	-
Zhang W et al. (2017) [7]	APP/PS1 transgenic mice	2 groups: 12 × NSC; 10 × WT	NSC	1 × 10^6^ cells/mL	increased neuron numbers	10 weeks	MWM/6 days	improvements in spatial acquisition
McGinley LM et al. (2018) [30]	APP/PS1 transgenic mice	3 groups: 10 × NSCs, 10 × sham operation, 14 × WT in 2 cohorts	NSC	1.8 × 10^5^ cells/mL	decreased amyloid plaque deposits, increased microglia activity	17 weeks	(1) MWM/5 days; (2) NOR/2 days	(1) improvements in spatial acquisition, (2) improved short-term non-associative memory
Hu W et al. (2019) [31]	chemical-induced rat model	4 groups: 6 × saline, 6 × no treatment, 6 × AD-CLN sham, 6 × AD-CLN-BDNF	BDNF-mhUC-MSCs-derived cholinergic-like neurons	2 × 10^5^ cells/mL	increased synaptic activity, decreased amyloid deposits	9 weeks	MWM/5 days	significantly reduced the escape latency
Li LY et al. (2008) [32]	chemical-induced rat model	5 groups: 5 × WT, 5 × AD, 5 × AD + PBS, 5 × AD + BMSC, 5 × AD + BMSC-NFG	BMSC-NGF	1 × 10^6^ cells/mL	prevented cell death, promoted neurogenesis, and was able to differentiate into cholinergic-like neurons	5 days	MWM/5 days	significant improvement in cognition in the BMSC or BMSC-NGF-treated groups
Zhang XM et al. (2020) [6]	chemical-induced rat model	4 groups: 25 × control, 25 × AD + PBS, 25 × AD + DPSC, 25 × PBS + PBS	DPSC	5 × 10^6^ cells/mL	increased neuron numbers	29 days	radial arm maze	decreased the total time required to accomplish the maze and the number of errors
Esmaeilzade B et al. (2012) [33]	chemical-induced rat model	4 groups: 10 × WT, 10 × AD, 10 × sham operation, 10 × EPI-NCSC	EPI-NCSC	2 × 10^5^ cells/mL	increased cell number and cell differentiation in glial cells: cholinergic neurons	4 weeks	Y-maze	no significant modification between the tested groups
Babaei H et al. (2023) [34]	chemical-induced rat model	5 groups: 8 × WT, 8 × sham operation, 8 × amyloid induced rats, 8 × AD with low dose, 8 × AD with high dose	MSC	50 × 10^4^ and 25 × 10^4^ cells/mL	improved oxidative stress, lowered neuroinflammation	58 days	MWM/5 days	the high-dose group, in comparison with the AD group, exhibited insignificant variation in the spatial learning function
Babaei P et al. (2012) [35]	chemical-induced rat model	2 groups: 10 × Ibo + MSC, 10 × Ibo + PBS	MSC	500 × 10^3^ cells/mL	increased cholinergic neurons	2 months	MWM/4 days	improvement in latency to the target quadrant; they did not reach the young group score
Babaei H et al. (2023) [36]	chemical-induced rat model	5 groups: 8 × WT; 8 × sham operation; 8 × amyloid induced rats; 8 × MSCs; 8 × MSCs + DMF	MSC with DMF promoter	25 × 10^4^ cells/mL	improved oxidative stress	58 days	MWM/5 days	reduction in escape latency time, also with DMF that boosted the efficacy
Cui GH et al. (2016) [37]	chemical-induced rat model	7 groups: 15 × WT, 15 × AD, 15 × NSC, 15 × SP, 15 × NSC + SP, 15 × DSP, 15 × NSC + DSP	NSC with SP or DSP	5 × 10^5^ cells/mL	increased neuron numbers	4 weeks	MWM/5 days	NSC + SP could decrease latency significantly; NSC + DSP had the shortest latency among the treated groups
Marei HE et al. (2014) [38]	chemical-induced rat model	4 groups: 16 × WT, 16 × lesioned group, 16 × lesioned with injection of vehicle, 16 × hNGF and OBNSC transplant	OBNSC	2.5 × 10^4^ cells/mL	increased cholinergic neurons prevented loss of neurons, induced new regenerative response in neurons	8 weeks	MWM	attenuated learning and memory impairment
Huang N et al. (2019) [39]	chemical-induced rat model	4 groups: 6 × PBS, 6 × AD, 6 × MSCs,6 × TIIA-MSCs	TIIA-MSCs	5 × 10^6^ cells/mL	increased neuron numbers	29 days	MWM/5 days	improved spatial learning and memory impairments in rats and are superior to MSC
Martinez-Losa M et al. (2018) [40]	hAPP-J20 transgenic mice	2 groups: 10–20 × MGE with NAV1.1.; sham operation	MGE (medial ganglionic eminence) with NAV 1.1. increased or decreased expression	0.5 − 1 × 10^6^ cells/mL	improved cognition and behavior	not mentioned	MWM/5–6 days	NAV 1.1. overexpression improved learning in the hidden platform
Fujiwara N et al. (2015) [41]	PDAPP transgenic mice	2 groups: 21 × hiPS; 19 × PBS	hiPS cell	2 × 10^5^ cells/mL	improved synaptic activity	41 days	MWM/6 days	mean platform escape latency of the transplanted mice was significantly shorter

ADSCs (adipose-derived mesenchymal stem cells), BDNF (brain-derived neurotrophic factor), BMSCs-NGF (bone marrow stem cells nerve growth factor), DPSC (dental pulp stem cell), DSP (designer self assembly peptide), EPI-NCSCs (epidermal neural crest stem cells), GFP-BMSCs (bone marrow stromal cells), hAM-MSCs (human amniotic mesenchimal stem cells), HBBS (hank’s balanced salt solution), hiNPCs (human-induced neural progenitor/stem cells), hNSCs (human neural stem cells), hUC-MSCs (human umbilical cord mesenchymal stem cells), HuCNS-SCs (human CNS-derived stem cells), Ibo (ibotenic acid), MSCs (mesenchimal stem cells), MSCs with DMF promoter (mesenchimal stem cells with dimethyl fumarate), MWM (Morris water maze), NOR (novel object recognition test), NSCs (neuronal stem cells), OBNSCs (olfactory bulb neural stem cells), OF test (open field test), PBS (phosphate-buffered saline), SP (self assembly peptide), WT (wild-type), TIIA-MSCs (tanshinone Iia incubated mesenchymal stem cells), hiPS cell (human-induced pluripotent stem cell).
